# Progress in Medicine

**Published:** 1896-05-02

**Authors:** 


### Progress in Medicine.

### DIABETES.

(*Continued from page* 58.)

Another important and interesting point is the relation between adiposity and diabetic glycosuria. He does not believe in the usual hypothesis that the stout patients are diabetic owing to the adiposity, but that this kind of adiposity is the result of diabetic disposition. This doctrine he explains by recalling the fact that usually in diabetes the tissues have lost not only their power of oxidising carbohydrates in the blood, but also their faculty of converting carbohydrate into fat. Were this not the case, the hyperglycæmia would obviously result, not in persistent glycosuria but in fat formation. It is conceivable, however, that the loss of oxidising power may predominate over the other defect. Consequently, the diabetes may be partially or even completely mastered. Cases of fatness where solely the oxidation of sugar is limited, but not its conversion into fat, he terms diabetogene adiposity.

The glycogenic function of the liver is universally admitted, but as regards the physiological destination of the glycogen there is some divergence. Pavy, it is well known, denies that the glycogen is normally converted into sugar, and brings forward evidence which he considers affords fair proof that it is converted into fat. It is beyond the scope of our review to weigh the evidence adduced by himself on the one one hand and Noel Paton, on the other, in favour or against the correctness of his contention. Noel Paton,[2] however, has recently pointed out that the liver does form fat from carbohydrate food, though it also forms sugar from the same source. Part of the liver-formed fat entered into the formation of lecithin, the source of phosphorus for the bone and the nucleins of the growing chick. Paton suggested that the store of fat on the liver was maintained to combine with the phosphorus produced by the decomposition of the nucleins, and to recombine for use again in the protoplasm. He showed that carbohydrate food increased the fats of the liver, and that as the glycogen disappeared there was an actual increase in the amount of fats. Since this increase was of the characteristic fats of the liver, and since the blood serum manifested no milkiness, he considered that the fats were actually formed by the liver protoplasm from the glycogen. Paton[3] further recalls some experiments made some time back by himself and Gulland, on the absorption of carbohydrates by the intestinal epithelium. One rabbit was fed on ordinary oats, another on oats deprived of fats by extraction with ether. One rabbit was fed on oats rich in fat; another on turnips poor in fat, but rich in carbohydrates. In both cases, while the rabbit fed on fat-containing oats had fat in the epithelium, the other, though perfectly healthy and vigorous, had none. Analogous experiments of feeding rats on lean horseflesh, on starch and sugar, and on ox-fat, respectively, proved that in the former no fat is present in the cells, while in the last there was abundance. Thus the fat is not formed from carbohydrates in the intestinal epithelium, and consequently it is practically certain it is formed in the liver when, as in patients or animals on carbohydrate diet, or diet containing no fat, fat is formed both in the liver and in the tissues elsewere. His experiments are particularly interesting, taken in conjunction with Noorden's remarks on "diabetogene adiposity." Pierre Marie[4] recognises many types of diabetes. In a recent clinical lecture he described several typical cases, amongst others of arthritic diabetes. He dwelt on the occurrence of gangrene and perforating ulcer in connection with diabetes, and remarks of one such case that it is a question whether it is due to certain vascular changes, like certain forms of senile gangrene, or to some peripheral or central nervous lesion. The coincidence in some cases of abolition of the knee-jerks and of perforating ulcer plead in favour of a nervous origin. But another theory attributes to local infection of the wound and subjacent tissues an important *rôle* in the development of the inflammatory or gangrenous symptoms. As Marie's patient suffered so greatly from his perforating ulcer, he had his leg amputated. Speaking on the question of surgical operations in diabetes, Marie says decisively that, without pretending to treat this question exhaustively, he believes there is no occasion for a medical man to dread beyond measure surgical intervention in diabetes, while, on the other hand, he must not be too confident of success. He remarks that it is obvious that the use of antiseptics, though greatly lessening the risk of diabetic patients dying from the effects of an operation, is far from being innocuous; for this reason there is a marked tendency among surgeons who are willing to operate on diabetic patients to have recourse to asepsis rather than to antisepsis.

Referring to the problems suggested by cases of conjugal diabetes, Marie remarks that at first sight one is prompted to regard such coincidences of husband and wife contracting diabetes as accidental. But in diabetes such coincidences are of too frequent occurrence to permit of explaining them away on the assumption of their being merely accidental. Cases of this kind were reported many years ago, at first merely as a curious coincidence; but as they became more and more numerous, the question began to be asked whether there was not a connection of cause and effect between these phenomena. Lecorché in his treatise reported six cases of conjugal diabetes among a total of 114 cases of diabetes under his observation; others were brought forward by Seegen and Betz. In 1889, Debove reported to the Medical Society of the Hospitals five cases of conjugal diabetes among fifty diabetic patients under his personal observation, and suggested the possibility of contagion between the husband and wife. This was followed by an important series of contributions to the question of conjugal diabetes. Rendu, E. Labbé, F. Dreyfous, and Gouraud reported each one case; Gaucher, Letulle, and Barthélemy had had two cases each under their care; Féréol had met with "several cases" among his patients. On the other hand, R. Schmitz has observed twenty-six cases of conjugal diabetes among 2,320 diabetic subjects. Adding to these three cases under the observation of Fouquet (Cairo), quoted by Le Gendre in his excellent article in the *Traité de Médécine*, we find in these hastily compiled statistics upward of 50 cases of conjugal diabetes.

These figures are certainly calculated to give cause for reflection, being in fact considerably higher than those in other conjugal affections in which contagion unquestionably plays an important *rôle*, viz., conjugal tabes, and conjugal general paralysis, syphilitic affections transmitted between consorts.

Marie goes on to say that two distinct theories have been advanced to explain the simultaneous occurrence of diabetes in husband and wife. Some authorities allege that the fact of married people living in the same manner and sharing the same food, the same anxieties, and the same sorrows, is sufficient to determine conjugal diabetes. According to the other theory, the phenomenon is the result of contagion. Marie inclines to the latter view, for the arguments that can be adduced in support of the contagion theory are the more plausible. Schmitz expressly states that in cases under his observation in which the husband or wife was secondarily affected, the patients, especially as regards women, had before been in excellent health, and had long been taking care of a diabetic patient, with whom they lived in the closest intimacy, sleeping in the same room, if not in the same bed. Teissier (Lyons) goes still farther and reports some striking cases of contagion between persons who were not in marital relations. One case was that of a laundress, who was said to have become diabetic, after having for six months washed the linen of a man and his little daughter who both suffered from well-marked diabetes. The second patient was a man whose mother had suffered from diabetes for many years, and who ultimately became diabetic himself. Six months later the family cook presented symptoms of diabetes. In addition to her other duties, this woman washed the pocket handkerchiefs of the master of the house. It is a still more curious fact that, a few months thereafter, a sewing-woman who for ten years had been in the habit of going to the same house to mend the linen and assist the cook, was also found to be diabetic. Teissier also reports two other cases in which persons living together developed diabetes. Thus, continues Marie, cases of conjugal diabetes therefore are comparatively frequent, and it is no longer possible to entertain doubts as to its occurrence. In all probability they are cases of genuine contagion. We are still at a loss to account for the exact manner in which this contagion takes place, though no doubt this question will be cleared up by future investigations. Charrin,[5] in his researches on experimental pancreatic diabetes of infective origin, has already made some progress in this direction.

Referring to the nervous phenomena associated with diabetes, Marie remarks that an attack of hemiplegia, as a complication of diabetes, presents the following special characters: (1) Comparatively rare occurrence of loss of consciousness, and especially of true apoplexy. (2) Irregularity of the paralytic phenomena; hemiplegic manifestations in diabetic patients are frequently peculiar in respect of their localisation or their intensity. Marie has met with Weber's syndrome, nearly isolated word-blindness, transitory hemianopsia, ocular paralysis, and monoplegia of the face or limbs. (3) Tendency to spontaneous retrogression.

[2] Edin. Med. Chir, Soc., Jan. 15, 1896; Brit. Med. Journ., Jan. 25, 1896. [3] Edin. Med. Journ., Feb., 1896. [4] Med. Week, Jan. 10, 1896. [5] Diabète pancréatique experimental d'originè infectieuse, Compte rendu des Congrès français de Méd. Int. de Lyon, 1894, p. 101; Med. Week, 1894. p. 532.

